# Carbon Materials in 2025: From Lightweight Structures to Sustainable and Multifunctional Platforms

**DOI:** 10.3390/ma19102020

**Published:** 2026-05-13

**Authors:** Stefano Bellucci

**Affiliations:** 1 Qi S.r.l., Via Monte d’Oro, 2/A, 00071 Pomezia, RM, Italy; stefano.bellucci@lnf.infn.it; 2 Nano Research Laboratory, Excellent Center, Baku State University, Academician Zahid Khalilov Street 33, AZ1148 Baku, Azerbaijan

## 1. Introduction and Scope

Carbon materials continue to occupy a singular place in materials science because they can be engineered across multiple length scales while retaining a rare combination of low density, high specific performance, chemical tunability, electrical functionality, and processing flexibility. From carbon fibers and graphene to activated biocarbons, diamond-like carbon coatings, and carbon-filled polymers, the field no longer advances through isolated improvements in a single property. Instead, the most significant work increasingly links composition, interface design, processability, and application-level performance. External reviews across thermoplastic composites, laser-induced graphene, biomass-derived carbons, graphene-enabled bioprocesses, and diamond-like carbon tribology all point in the same direction: carbon materials are becoming less a narrow class of “advanced fillers” and more a platform technology for structural efficiency, sustainable processing, and multifunctional design. This broader trajectory is well documented in work on graphene [1], laser-induced graphene [2,3], carbon-fiber-reinforced thermoplastic composites [4,5,6], biomass-derived carbons [7,8], graphene-enabled bioprocesses [9,10], and diamond-like carbon tribology [11,12].

The seven Editor’s Choice Articles selected in the Carbon Materials Section of *Materials* in 2025 illustrate this shift particularly well. Considered together, they span lightweight structural composites, porous carbon adsorbents, deformation-aware conductive polymers, laser-induced graphene, graphene-assisted anaerobic digestion, hard carbon-based tribological coatings, and self-lubricating carbon-modified resins. Their scientific value lies not only in the quality of the individual studies, but also in the collective picture they provide of where carbon materials research is moving.

Three features stand out. First, structure–property relationships remain central, but are increasingly studied under realistic processing and service conditions rather than in idealized laboratory form. Second, sustainability is no longer a peripheral theme: biomass-derived carbons, process simplification, reduced part weight, and performance in environmental systems are becoming core design criteria. Third, multifunctionality is now expected. Mechanical reinforcement alone is not enough; a carbon-based system is increasingly valued for simultaneously enabling sensing, lubrication, adsorption, and process intensification.

## 2. Emerging Directions in the Selected Articles

A first direction highlighted by the selected papers is the growing maturity of carbon materials for lightweight structural applications. The study by Min et al. [Contribution 1] on the injection molding of a firefighter back-rest component is a strong example of engineering translation. Rather than presenting carbon-fiber reinforcement in abstract terms, the authors addressed a specific and demanding use case in personal protective equipment, combining long-fiber thermoplastic processing, flame-retardant formulation, and process optimization. By using a PA66-based long-fiber thermoplastic with carbon fiber and optimizing the molding conditions through a Taguchi approach, the work achieved a meaningful reduction in component weight while maintaining the required mechanical performance. This is the kind of study that matters for the next phase of carbon materials research: not simply whether a material can be stronger or lighter in principle, but whether it can be manufactured reproducibly in a geometry, process window, or regulatory context that matters in practice. These translational aspects are fully aligned with the broader literature on carbon-fiber-reinforced thermoplastics and their manufacturing pathways [4,5,6].

A complementary perspective on carbon-filled structural polymers is provided by Solberg et al. [Contribution 2], who investigated tensile deformation and transverse strain behavior in carbon black–UHMWPE composites. The importance of this work lies in its attention to geometric evolution during large plastic strain. Carbon-containing polymers are often discussed for their conductive or reinforcement functions, but real applications such as wearable devices, impact monitoring, orthopedics, and high-strain sensing depend on how the material deforms volumetrically, necks locally, and departs from ideal assumptions such as volume conservation. By quantifying these deviations, the authors established a more realistic basis for linking mechanical strain to electrical or functional response. This is a valuable reminder that multifunctionality requires careful mechanics: a conductive composite is useful only insofar as its evolving geometry, filler network, and structural integrity are understood together.

A second major direction emerging from the selected articles is sustainable carbon processing. In this respect, the chestnut-based biocarbons reported by Charmas et al. [Contribution 3] are particularly significant. Using horse chestnut biomass and physical activation with carbon dioxide or steam, the authors showed how low-cost plant-derived precursors can be transformed into porous adsorbents with relevant structural and adsorption properties. Equally important, the work addressed contaminants of clear environmental interest, including tetracycline, naproxen, and methyl orange. This paper reflects a broader shift in carbon materials research: biomass-derived carbons are no longer regarded only as low-cost substitutes for conventional activated carbons, but as tunable adsorbent systems whose surface chemistry, pore architecture, and preparation route can be tailored to specific classes of pollutants. The scientific challenge moving forward will be to connect precursor chemistry, activation route, and sorption selectivity more predictably, while also addressing regeneration, water-matrix effects, and scale-up. This wider context is consistent with recent reviews on biochar and biomass-derived activated carbons for water treatment and pollutant removal [7,8].

The environmental theme appears again, but in a very different form, in the work of Usevičiūtė et al. [Contribution 4] on graphene-based nanomaterials in the anaerobic digestion of thermally hydrolyzed municipal sewage sludge. This study is notable because it places carbon nanomaterials inside a complex biological and process-engineering environment rather than a conventional solid-state materials setting. By comparing graphene nanoplatelets of different surface areas and graphene oxide, the authors examined how carbon nanostructures can influence methane production and digestion kinetics. This is an important frontier. Carbon materials are increasingly being explored not only as passive supports or sorbents, but as active mediators of interfacial transport, microbial interactions, and process intensification. At the same time, this area requires caution. The promise of performance enhancement must be balanced against questions of reproducibility, long-term stability, dosage dependence, and the broader ecological implications of introducing graphenic additives into environmental technologies. The significance of the paper lies precisely in helping move this topic from speculation toward measurable process understanding. More broadly, this topic connects with recent reviews on graphene oxide and graphene-based nanomaterials in anaerobic digestion and microbial systems [9,10].

A third direction highlighted by the 2025 selection is the continued expansion of carbon materials as multifunctional surfaces and interfaces. The paper by Botti et al. [Contribution 5] on CO_2_- and UV-laser-induced graphene from polymer precursors captures this very well. Laser-induced graphene has become one of the most dynamic areas in applied carbon materials because it combines direct writing, local patterning, and scalable polymer-to-carbon conversion. Detailed comparison between UV and CO_2_ laser processing is therefore especially valuable. By combining two-dimensional Raman mapping with microscopy, the authors showed that the more-ordered carbon produced by CO_2_ processing does not simply make UV-derived material obsolete; rather, UV processing offers distinct advantages in patterning resolution and reduced penetration depth. This kind of processing–structure trade-off is central to the maturation of laser-induced graphene. The field is moving beyond the simple demonstration that LIG can be produced, toward a more application-driven question: which laser, substrate, and irradiation regime produce the kind of graphitic architecture needed for a given sensing, electronic, thermal, or surface-engineering function? These observations are also consistent with the rapidly expanding laser-induced graphene literature [2,3].

Tribology is another area in which the selected Editor’s Choice papers show the breadth of carbon materials innovation. Yang et al. [Contribution 6] reported on a diamond/diamond-like carbon composite coating for dry environments, demonstrating improved tribological behavior relative to conventional reference coatings. Carbon-based tribological systems remain strategically important because they address a problem of great technological and environmental significance: reducing friction and wear without relying on conventional liquid lubricants. The appeal of diamond and DLC systems has long been recognized, but the practical challenge has always been to combine hardness, adhesion, low friction, and environmental robustness in a single architecture. Composite coating strategies are therefore especially important because they use carbon not as a single ideal phase, but as part of a deliberately engineered surface system. This is a recurring motif across modern carbon materials research: performance arises from hybridization and interface control more often than from material purity alone. The broader tribological context is well established in classic and recent reviews of diamond-like carbon films [11,12].

The same principle is visible in the paper by Warycha et al. [Contribution 7] on carbon-modified polydicyclopentadiene (PDCPD) resin. Their comparison of carbon nanotubes, carbon fibers, and graphite fillers makes a useful point that is sometimes obscured in the nanomaterials literature: the “best” carbon additive depends strongly on the targeted function and the compatibility with the host matrix. In this study, graphite and carbon fibers provided the most favorable balance of mechanical support and friction reduction, whereas carbon nanotubes reduced strength substantially, indicating poor matrix compatibility under the chosen conditions. This negative result is scientifically valuable. Carbon materials research advances not only through the celebration of high-performing nanostructures, but through careful identification of where a popular filler does not perform as expected. Such studies help move the field away from generic assumptions and toward a more disciplined, application-specific selection of filler type, morphology, loading, and processing route. A useful comparison is provided by recent work on graphite-filled self-lubricating epoxy composites [13].

Taken together, the seven selected papers indicate that carbon materials research is increasingly defined by convergence. The field now links structural composites with environmental engineering, tribology with lightweight design, and advanced characterization with manufacturing-aware optimization. This convergence is not accidental. Carbon materials are uniquely positioned to bridge molecular-scale tailoring and macroscopic function. But the 2025 selection also shows that future progress will depend less on isolated demonstrations of exceptional properties and more on the integration of four criteria: scalable processing, stable interfaces, multi-function performance, and application-relevant testing conditions.

## 3. Conclusions

The seven Editor’s Choice Articles discussed here offer an instructive snapshot of the current state of carbon materials research. They show a field that is broad in application but increasingly coherent in scientific direction. Whether the goal is to lighten a structural component, create a low-cost porous adsorbent, understand the deformation of conductive polymers, pattern graphene directly from polymers, enhance anaerobic digestion, reduce friction under dry conditions, or engineer a self-lubricating polymer composite, the same general principle emerges: carbon is most powerful when its structure, interface chemistry, and processing pathway are designed together.

This is why these papers are significant beyond their individual results. They collectively demonstrate that carbon materials are not maturing into a closed field with fixed material classes and standard applications. On the contrary, they are becoming more versatile, more application-aware, and more deeply integrated into sustainability-driven engineering. Future advances will likely come from studies that combine multi-scale characterization with data-guided process optimization, quantify long-term durability and life-cycle impact, and treat carbon materials not as isolated phases but as functional systems embedded in real devices, processes, and environments.

In this sense, the Carbon Materials Section of *Materials* is well positioned to capture one of the most important developments in contemporary materials science: the transformation of carbon from a celebrated class of high-performance materials into a practical foundation for lightweight, sustainable, and multifunctional technologies.
